# Vaccines for Protecting Infants from Bacterial Causes of Diarrheal Disease

**DOI:** 10.3390/microorganisms9071382

**Published:** 2021-06-25

**Authors:** Richard Walker, Robert W. Kaminski, Chad Porter, Robert K. M. Choy, Jessica A. White, James M. Fleckenstein, Fred Cassels, Louis Bourgeois

**Affiliations:** 1 Center for Vaccine Innovation and Access, PATH, Washington, DC 20001, USA; lbourgeois@path.org; 2 Department of Diarrheal Disease Research, Walter Reed Institute of Research, Silver Spring, MD 20910, USA; robert.w.kaminski.civ@mail.mil; 3 Enteric Diseases Department, Naval Medical Research Center, Silver Spring, MD 20910, USA; chad.k.porter2.civ@mail.mil; 4 Center for Vaccine Innovation and Access, PATH, San Francisco, CA 94108, USA; rchoy@path.org; 5 Center for Vaccine Innovation and Access, PATH, Seattle, WA 98121, USA; jawhite@path.org (J.A.W.); fcassels@path.org (F.C.); 6 Department of Medicine, Washington University School of Medicine, St. Louis, MO 63110, USA; jfleckenstein@wustl.edu; 7 Medicine Service, Saint Louis VA Health Care System, St. Louis, MO 63106, USA

**Keywords:** multi-pathogen enteric vaccines, *Shigella* vaccine, ETEC vaccine, *Campylobacter* vaccine, mucosal immunity, disease burden, stunting, models of disease, adjuvants

## Abstract

The global diarrheal disease burden for *Shigella*, enterotoxigenic *Escherichia coli* (ETEC), and *Campylobacter* is estimated to be 88M, 75M, and 75M cases annually, respectively. A vaccine against this target trio of enteric pathogens could address about one-third of diarrhea cases in children. All three of these pathogens contribute to growth stunting and have demonstrated increasing resistance to antimicrobial agents. Several combinations of antigens are now recognized that could be effective for inducing protective immunity against each of the three target pathogens in a single vaccine for oral administration or parenteral injection. The vaccine combinations proposed here would result in a final product consistent with the World Health Organization’s (WHO) preferred product characteristics for ETEC and *Shigella* vaccines, and improve the vaccine prospects for support from Gavi, the Vaccine Alliance, and widespread uptake by low- and middle-income countries’ (LMIC) public health stakeholders. Broadly protective antigens will enable multi-pathogen vaccines to be efficiently developed and cost-effective. This review describes how emerging discoveries for each pathogen component of the target trio could be used to make vaccines, which could help reduce a major cause of poor health, reduced cognitive development, lost economic productivity, and poverty in many parts of the world.

## 1. Introduction

The development of a vaccine against *Shigella*, a major cause of bacterial dysentery, was pursued in the early part of the 20th century, and in the 1960s, new approaches to develop genetically attenuated *Shigella* began [1]. Since then, numerous other enteric pathogens have also been associated with infectious diarrhea [2,3,4]. With exceptions such as cholera and rotavirus, there are still no licensed vaccines against many enteric pathogens. This is true for *Shigella* and for the more newly recognized pathogens such as enterotoxigenic *Escherichia coli* (ETEC) and *Campylobacter jejuni*, which have been identified as major causes of enteric disease. Nevertheless, significant progress has been made towards vaccine development for some of these pathogens, such that effective and practical vaccines to prevent a major portion of the diarrheal disease they cause could soon be reached. Recent advances in the vaccine pipelines for both *Shigella* and ETEC led the WHO to recently reaffirm these pathogens as priority vaccine targets and to develop preferred product profiles (PPCs) for both vaccines. The WHO has also urged that combination vaccine approaches be considered, as this option may improve the full public health value proposition for these vaccines and therefore improve the prospects for more widespread uptake in low- and middle-income countries (LMICs).

## 2. *Shigella*, ETEC and *Campylobacter*: Targets for Vaccine Development

**Figure 1:**
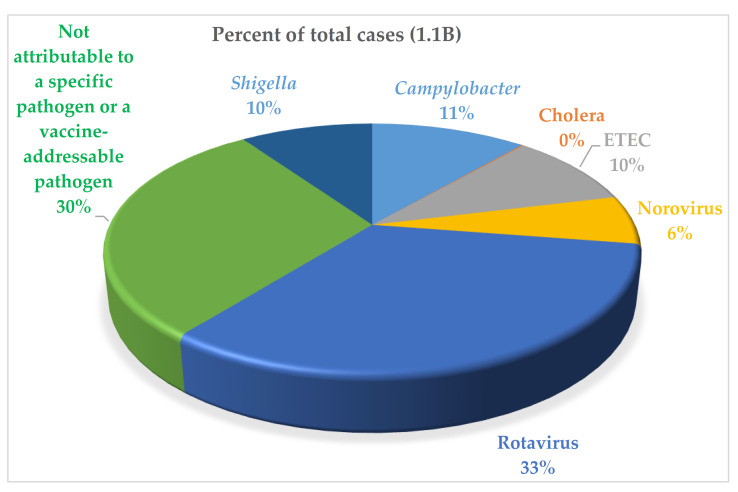
Percent of total cases of diarrheal disease caused by specific pathogens. Cholera shows as negligible in the chart because of the low number of cases compared with the other causes of enteric diseases shown. These are data for 2016 cited in [5].

The Institute for Health Metrics and Evaluation (IHME) estimates demonstrate that *Campylobacter*, *Shigella*, and ETEC, in spite of regional variations in distribution [5], account for one third of the global diarrheal disease burden with estimates of 88M, 75M, and 75M cases annually, respectively (Figure 1) [5,6,7]. According to the IHME estimates, *Shigella* accounts for the highest percentage of deaths among enteric bacterial pathogens (14%), followed by *Campylobacter* (9%) and ETEC (4%). In addition to acute diarrheal disease, infections with these three pathogens are also associated with both physical and intellectual stunting in children as well as other long-term sequelae of enteric infection, including reactive arthritis, Guillain-Barre Syndrome, and an increased risk of mortality due to other infectious diseases in stunted children [8,9,10]. These three pathogens are also designated as antimicrobial resistance threats by the WHO; therefore, vaccine development is being prioritized for all three pathogens [11,12]. A vaccine against this target trio of enteric pathogens could address about one-third of diarrhea cases in children. Rotavirus accounts for another third. The final third of diarrheal cases may include members the target trio not identified or other organisms for which no vaccine candidates are on the horizon.

It is possible that a tri-pathogen vaccine against *Shigella*, ETEC, and *Campylobacter* could be realized relatively quickly if current progress could be directed and funded towards vaccines exploiting conserved antigens (see discussions under individual pathogens below) providing broad coverage against multiple pathogens. There is an urgency for availability of these vaccines in LMICs, and it is the central thrust of this review article to consider options to develop them. In recent years, the major driver for enteric vaccine development has evolved from a primary focus on reducing mortality to now also factoring in reductions in acute and more longer-term morbidity, as well as socio-economic benefits. This approach will be more formally defined in value proposition documents that are under development for both *Shigella* and ETEC. The advantage of a combination vaccine is well established for the pentavalent Expanded Program on Immunization (EPI) vaccines for diphtheria−tetanus−pertussis, *Haemophilus influenzae* type b, and hepatitis B, so a combination vaccine targeting multiple enteric pathogens may offer similar value. This review article describes how emerging discoveries regarding each pathogen component of the target trio could be leveraged to make combined products that could help reduce a major cause of poor health, mortality, reduced growth and cognitive development, lost economic productivity, and poverty in many parts of the world.

## 3. *Shigella* Component 

### 3.1. Serotype-Dependent Candidates

To date, *Shigella* vaccine research has been primarily focused on serotype-specific O polysaccharides (O-PS), although some preclinical work has also evaluated protein antigens that are more broadly conserved and also contribute to protection. An optimal *Shigella* vaccine would protect against *S. flexneri* 2a, 3a, and 6, as well as *S. sonnei*, which together account for over 80% of cases [13,14]. A vaccine focused only on O-PS would inherently need to incorporate O-PS from each serotype. This number of O-PS may be reduced with a possible increase in vaccine efficacy if more conserved antigens are exploited, which now seems possible. For example, a core *Shigella* proteome microarray consisting of over 2000 antigen targets common to all *Shigella* species was used to assess the serum samples from volunteers immunized with killed, attenuated, and wild-type *S. flexneri* 2a [15]. These studies identified a protein type three secretion system (T3SS) signature with antibodies against IpaB, IpaD, IpaA, IpaH, and IpaC being associated with clinical protection. In more recent proteomic and lipopolysaccharide array analyses, antibody levels against both *S. sonnei* LPS and the IpaB invasion protein were associated with a reduced risk of developing shigellosis and a reduced disease severity score following infection with the 53G strain [16,17]. Table 1 provides a summary of the development status of current *Shigella* vaccine candidates based on conserved proteins (most subunit candidates), serotype-specific O-antigens (glycoconjugate candidates and GMMA), or both types of antigens (cellular candidates). 

Approaches involving pathogen attenuation have historically dominated the field for *Shigella* vaccine candidates. Two current attenuation strategies for *Shigella* are a VirG-based mutant [18] and a guaBA-based mutant [19]. Further attenuation of these organisms was obtained through the deletion of *Shigella* enterotoxins 1 and 2 (ShET1 and ShET2). These newer attenuated *Shigella* vaccine candidates induce robust immune responses and have a safety profile superior to those seen with previous constructs [18,19]. A modification of the live attenuated *Shigella* strategy has been the use of the typhoid vaccine Ty21a as a vector for the major *Shigella* O-PS [20,21]. This vector has been made more stable than earlier versions and has been protective in animal models [20].

In contrast with attenuation, immunization with inactivated *Shigellae* has received little attention. The safety and ease of formulation of cellular *Shigella* vaccines may be further improved by the use of inactivated whole cell vaccines. This approach has been protective in animals [22], and inactivated *S. sonnei* and *S. flexneri* 2a vaccine prototypes were safe and immunogenic in Phase 1 trials [23,24]. As with the attenuated strategies described above, the inactivated whole cell approach relies on including strains to cover the major O-PS antigens, although responses to conserved antigens would also be present.

Extensive research has been conducted on subcellular approaches involving the intramuscular administration of O-PS conjugated to protein carriers. *Shigella* conjugate vaccines are safe and protective in adults and older children, but one prior conjugate has been shown to not be protective in children less than 3 years of age [25]. Recently, a recombinantly produced glycoconjugate candidate was tested in a Phase 2b clinical trial in adults that demonstrated moderate protection against more severe shigellosis following challenge [26,27,28]. A conjugate generated through a carbohydrate chemical synthesis approach is also in early clinical studies [29,30]. Whether the immunogenicity and protection reported with conjugates in adults are due to boosting previous mucosal exposure to the O-PS antigen or to the initiation of a predominantly systemic response remains to be determined. Hartman [31] found that reductions in severity following immunization with a conjugate vaccine from that seen with nonvaccinated animals were only obtained in cases when the vaccine regimen contained a priming mucosal immunization with EcSf2a-2, a live attenuated *Shigella* vaccine candidate [31]. When a parenterally administered *S. flexneri* 2a O-antigen conjugate vaccine was given alone, infection severity was essentially identical to that seen in the nonimmunized control animals. In a similar vaccine approach to the O-PS conjugates, outer membrane vesicles of *Shigella* generated by the Generalized Modules for Membrane Antigens (GMMA) approach are being developed as O-PS vaccines [32,33]. The double-mutant heat-labile toxin (dmLT) adjuvant given parenterally helps direct a mucosal response as well as the systemic immunity ordinarily obtained by this route [34]. This property of dmLT may benefit the immunogenicity of the conjugate vaccines, GMMA, and other parenterally administered vaccines. In addition, recent Phase 1 and preclinical studies indicate that dmLT can improve the serum and mucosal antibody responses to LPS antigens, which might further enhance its benefit for candidate *Shigella* vaccines [35].

### 3.2. Serotype-Independent Candidates

In addition to O-PS-based vaccines, work is underway to exploit conserved virulence proteins that may provide broad serotype-independent coverage against *Shigella*. T3SS proteins involved in cellular invasion have shown broad protection in animals. For example, invasion plasmid antigen (Ipa) proteins such as IpaB and IpaD have protected mice against lethal challenge from multiple serotypes of *Shigella* [36,37,38,39].

The invasion complex or Invaplex is an example of a subunit vaccine that is a partially serotype independent approach that incorporates the serotype specific LPS into a macromolecular complex with broadly conserved Ipa proteins. First-generation Invaplex vaccine candidates were isolated from water extracts of virulent shigellae [40,41] and were shown to be safe and immunogenic in clinical studies [42,43]. In an effort to increase immunogenicity, refine the manufacturing process, and further optimize the concept, efforts have focused on the development of an artificial Invaplex product, with the complex assembled from recombinant IpaB, IpaC, and purified LPS [44]. Further refinements of the Invaplex product, which utilize LPS with under-acylated Lipid A, have shown robust immunogenicity and efficacy in several preclinical models when delivered intramuscularly [45]. More importantly, the artificial detoxified Invaplex (Invaplex _AR-Detox_) vaccine candidate delivered intramuscularly without an adjuvant has been shown to be safe, well-tolerated, and highly immunogenic in a recently completed Phase 1 clinical study (NCT03869333), justifying the further evaluation of this approach. The vaccine induced serum antibody responses directed to the three major vaccine constituents (LPS, IpaB, and IpaC) in 80–100% of subjects across the three dose cohorts (2.5, 10, and 25 µg). Moreover, the serological responses were found to be highly functional, with bactericidal responses that exceeded those induced after experimental oral infection with *S. flexneri* 2a, 2457T. The preliminary results also indicate that the bactericidal activity is not limited to *S. flexneri* 2a, but extends to other *S. flexneri* serotypes responsible for global morbidity and mortality, likely attributable to the high levels of antibodies directed to the broadly conserved Ipa proteins. ALS IgG and IgA titers from α4β7+ PBMC populations after immunization with Invaplex_AR-DETOX_ exceeded those induced after oral infection with *S. sonnei*, 53G or *S. flexneri* 2a, 2457T in CHIM studies [16,28]. The frequency and magnitude of the mucosal immune responses was encouraging, given the parenteral route of vaccine delivery.

A surface polypeptide (IcsP) located on the *Shigella* virulence plasmid harbors a prominent cross-protective moiety, pan *Shigella* surface protein 1 (PSSP-1), common to over 300 *Shigella* strains tested [46,47]. Mucosal administration of cholera toxin- or dmLT- adjuvanted PSSP-1 induces broad protection in mice against experimental challenge, with strains belonging to all major species and serotypes of *Shigella* [46]. This candidate proved difficult to scale up for the preparation of clinical lots and was deprioritized.

Two novel whole cell approaches are now being pursued that seek to exploit more conserved antigens of *Shigella*. One builds on the PSSP1 antigen strategy in that it involves the unmasking of surface antigens such as PSSP1 on the whole cell by limiting the length of polysaccharide chains synthesized to a single repeating unit. This is the *Shigella* Truncated Mutant (STM), which utilizes genetically modified (Δwzy) inactivated bacteria retaining only one repeating unit of O antigen chain on the bacterial surface [48]. The other candidate, ShigETEC, is attenuated by the deletion of the T3SS and is engineered to not express any LPS-O antigens through a targeted deletion of the rfbF gene [49,50,51]. Both of these novel approaches increase exposure of conserved outer membrane proteins and could effectively provide a broad coverage *Shigella* vaccine with a single cell type. Both may provide exposure of the PSSP1 protein, but only the STM would also retain and present potentially protective proteins of the T3SS. In addition, there may be other novel proteins on the surface of these “unmasked” constructs that could contribute to protection [15].

## 4. ETEC Component

Most ETEC vaccine candidates currently under development use cellular or subunit-based vaccine approaches and focus on the induction of anti-labile toxin (LT) and anti-colonization factor/coli surface (CF/CS) antibodies (Table 2), thereby blocking adherence to the intestinal lining and subsequent enterotoxicity. Cellular vaccine candidates against ETEC have included ACE527 (live attenuated) and ETVAX (inactivated whole cell). ACE527, consisting of three ETEC strains expressing major CF and CS antigens, as well as the B subunit of labile toxin, was significantly protective in people when co-administered with a non-toxic double mutant of LT, dmLT, which acts as a mucosal adjuvant and an antigen (see Section 7.2) [52]. This candidate is not currently under active development as an ETEC vaccine because of lack of funding. In contrast, ETVAX is undergoing active development, having recently completed a successful clinical trial in Bangladeshi adults and infants [53,54] and a protection trial in Finnish travelers to Benin [55]. This vaccine consists of four *E. coli* preparations engineered to express large quantities of the major clinically relevant colonization factors (CFA/I) and coli surface proteins designated CS3, CS5, or CS6. It is formulated with the B subunit of the cholera toxin modified to have stronger homology with the ETEC labile toxin [56]. The vaccine is co-administered with dmLT as an adjuvant.

A vaccine consisting of four strains of *Shigella* attenuated by deletion in the guaBA 196 operon (see above under *Shigella* component) wasconstructed as a *Shigella*-ETEC bivalent hybrid vaccine expressing major CFA/ICS antigens of ETEC, along with labile toxin B subunit (LTB) and a stable toxin (ST) toxoid [57]. A prototype of this vaccine, CVD1208S-122, was recently tested in an antibiotic treated mouse model and protection against disease was seen following oral challenge with ETEC or *Shigella* [58].

ShigETEC, the O-PS free *Shigella* vaccine construct described above, could also be a combined *Shigella*−ETEC vaccine, as it has been engineered to express toxin antigens for the LT and stable toxin (ST) of ETEC [49,50,51]. However, instead of CFA/I-CS antigens, this candidate relies on the homology between *Shigella* and ETEC surface proteins. *Shigella* and ETEC are phylogenetically related and share a 70% nucleotide similarity [59,60]. Whether the homology between the two organisms will be sufficient with antitoxin immunity against all major clinical strains of ETEC is unknown. However, some data reported by Medeiros et al. [58] indicate that at least some protection against ETEC is obtained with the CVD1208S strain not expressing CFs, suggesting some level of conserved protection in mice from *Shigella* antigens.

Other ETEC vaccine candidates based on subunit CFA/I-CS antigens, toxins, or novel antigens are also under development, as follows:

1. Immunity against CFA/I-CS antigens could block pathogenesis by interrupting the adhesion of the pathogen to the intestinal epithelium. An innovative, subunit ETEC candidate uses recombinantly produced conserved subunits of some CFA/I-CS proteins. These are the fimbrial tip adhesin (FTA) proteins from ETEC, which can induce strong immune responses at systemic and mucosal sites when co-administered intramuscularly with dmLT [61]. Individual FTA antigens, which would comprise a complete quadrivalent vaccine, have each protected non-human primates [62,63] vaccinated intramuscularly and challenged orally. Two of the FTA antigens have undergone Phase 1 evaluations. The first is CfaE, which was found to safe and immunogenic when given intradermally with mLT, and reduced the incidence and severity of disease following challenge with a CFA/I-expressing ETEC [55]. The inclusion of the mLT adjuvant in the vaccine significantly improved the serum IgA and IgG response to CfaE, as well as the HAI antibody response to CfaE adhesin [55,64]. The safety and immunogenicity of the CssBA (CS6) antigen with or without dmLT given intramuscularly have also recently been evaluated in a Phase 1 trial. The CssBA antigen was found to be safe up to a dose of 45 ug (highest dose tested), and the serum and mucosal antibody responses to the antigen were significantly improved by the addition of dmLT, including increased levels of anti-CS6 α4β7 cells in the peripheral blood and anti-CS6 fecal antibody levels [65,66].
2. Multiple epitope fusion antigens (MEFA) utilize CFA/I as a platform to express the dominant epitopes of other CFAs in a single protein, along with non-toxic LTA-LTB and ST [67,68]. MEFA vaccines stimulate neutralizing antibodies against the selected virulence antigens and piglets immunized with the MEFA–K88ac vaccine remained healthy following challenge [69,70]. Quantitative culture of the piglet ileum showed reduced colonization following immunization and K88 challenge. This candidate also protects rabbits against colonization by a human ETEC strain (ETEC strain B7A) [71] and could be cloned into a vector or delivered parenterally as a purified subunit.
3. The application of new “Omics” technologies and other gene-based approaches also offer great promise for yielding new vaccine antigens from ETEC that may provide broad protection, as well as to facilitate combined vaccine strategies [72,73,74]. Pangenome analysis of multiple strains, combined with open-aperture ETEC immunoproteome interrogation of samples from both human volunteers [72] and naturally infected hosts, indicate that there are relatively few, highly conserved, strongly immunogenic, and ETEC pathovar specific antigens. These studies have highlighted two antigens, EatA and EtpA. EtpA is an extracellular adhesin of ETEC that promotes bacterial attachment and toxin delivery [73], acting as a bridge between bacteria and human A blood group expressed on the intestinal epithelia [74]. Similar to EatA, vaccination with EtpA effectively reduces the intestinal colonization of mice by ETEC. An additional antigen and virulence factor found in ETEC, as well as in other diarrheagenic and extra-intestinal *E. coli*, is YghJ (sometimes designated SslE). This protein antigen has shown protection in animal models, suggesting that it may have a vaccine potential [75,76,77,78]. The inclusion of these non-canonical antigens in ETEC vaccine candidates could complement or broaden the protection afforded by CF-based antigens.
  The majority of ETEC strains express a mucin-degrading serine protease autotransporter protein known as EatA [79], and EatA expression was recently shown to correlate strongly with symptomatic infection among young children in Bangladesh [80]. SepA, discovered as the major secreted protein present in culture supernatants of *S. flexneri* 5a [81], is an orthologue of EatA with which it shares a ~75% amino acid identity. Surveys of *Shigella* genomes reveal the presence of either SepA or EatA genes in each *Shigella* species [82], with SepA predominating in *flexneri*, and EatA more commonly represented in *S. sonnei*. The secreted EatA passenger domain is strongly immunogenic following ETEC infection of humans [83], and vaccination with this domain reduces colonization of mice after challenge with ETEC [72]. Whether vaccination with EatA or SepA can afford cross protection against ETEC and *Shigella* in humans remains to be determined.
4. The virulence of ETEC strains is associated with LT and ST enterotoxins expressed in the small intestine. Toxoids based on the LT or ST antigens may be found to contribute to the effectiveness of ETEC vaccines. A transdermal LT patch, no longer in development, was used to show protection against LT-only producing strains in a Phase 3 trial [84]. dmLT has also been shown to induce anti-LT antibody responses in human volunteers and to induce better LT toxin neutralization antibody responses than when B-subunit toxoid preparations have been used [85]. Field and controlled human infection model (CHIM) study data indicate that inducing anti-LT immunity can be an immune marker for reduced risk of ETEC illness, particularly as a result of the ETEC strains expressing only the LT toxin [84,86]. CHIM and field studies also indicate that strong immune responses to LT can help to reduce the severity of ETEC illness when it occurs [84,87]. LT appears to promote ETEC colonization by changing the surface architecture of the intestinal epithelia to favor the pathogen attachment [88], and anti-LT immunity can cooperatively impact effective ETEC small intestinal colonization when combined with anti-adhesin approaches [73]. Moreover, LT has been shown to accentuate the enterotoxic effects of the ST toxin [89], suggesting that anti-LT immunity could be beneficial in mitigating the impact of both toxins. The importance of ST in a vaccine is less well established, but passive immunization studies in piglets have shown protection against disease [70]. Recent work has shown that the ST molecule can be detoxified without losing neutralizing properties, be made immunogenic by linking to a protein, and avoid cross reaction with human guanylin-uroguanylin [90,91,92]. In addition, novel studies of ST toxin secretion and delivery have shown that the STH propeptide is secreted by ETEC, potentially providing additional epitopes for inducing toxin neutralizing antibodies [93]. These studies also showed that EtpA adhesin plays an important role in ST toxin delivery to intestinal epithelial cells, suggesting that antibodies against this potential vaccine antigen could reduce ST toxicity [93].

## 5. Campylobacter Component

Several antigens of *Campylobacter* have been considered for vaccines, yet few candidates for *C. jejuni* vaccines are currently under development (Table 3) [94]. A prototype monovalent capsular polysaccharide (CPS) conjugate vaccine using CRM197 as the protein carrier has been evaluated in a number of preclinical studies, and was found to be highly immunogenic in mice, and demonstrated efficacy against diarrheal disease in *Aotus nancymaae*, a new world owl monkey species [95]. A Phase 1 first-in-human trial (ClinicalTrials.gov Identifier: NCT02067676) was recently completed demonstrating the safety and immunogenicity of the vaccine candidate [94]. In follow-on preclinical studies, the immunogenicity of the prototype CPS vaccine could be improved by administering it intramuscularly in a liposome carrier also containing MPL and QS-21. This adjuvanted vaccine candidate was protective in the new Zn-deficient *Campylobacter* mouse model [96]. It is anticipated that a complete vaccine against *Campylobacter* based on CPS would be multi-valent, which could raise manufacturing questions about its practicality for use in LMICs, particularly if part of a combination vaccine.

Three approaches utilizing highly conserved antigens have been explored as candidate vaccines for immunization against *Campylobacter*. One of these is flagellin [94], the immunodominant antigen recognized during infection with *Campylobacter.* Antibodies against flagellin correlates with the development of protection against disease [97,98,99,100,101]. Early CHIM studies demonstrated that volunteers challenged with *C. jejuni* strain 81–176 developed a robust immune response against the flagella [97]. In addition, the immunological response against the flagellin was correlated with the protection against disease during re-challenge studies [97]. To generate a recombinant flagellin-based vaccine, the conserved region of FlaA from *C. coli* VC167 was fused with the maltose binding protein (MBP) of *E. coli*. The vaccine (rFlaA-MBP) was formulated with mLT and was tested for immunological response and protective efficacy in a mouse colonization model. Encouraging results were obtained; an intestinal secretory IgA response and protection against heterologous colonization and disease by *C. jejuni* 81–176 were established when the rFlaA-MBP was adjuvanted with a single mutant of LT (LTR192G). This vaccine candidate progressed through a Phase 1 trial, but was abandoned because of poor immunogenicity, despite showing promising efficacy in mice [97]. The human trial, in contrast with prior animal studies, did not include the mLT adjuvant used in the mouse studies as a result of safety concerns associated with the intranasal administration of the mLT. This candidate could be reevaluated with a different route of administration to include an adjuvant with an established improved safety profile such as dmLT.

The remaining two antigens are in preclinical development. One is based on the homology between cholera toxin B subunit (CTB) and a 53 KDa major outer membrane protein, PorA of *Campylobacter*. Immunization with CTB reduced the colonization of adult mice challenged with *C. jejuni* [102,103]. The other approach utilizes a conserved *N*-glycan heptasaccharide of *Campylobacter* for immunization. This antigen was displayed on *E. coli* to immunize chickens and provided up to a 10-log reduction in *C. jejuni* colonization following challenge [104]. This effect was also obtained in chickens with a the heptasaccharide conjugated to an engineered *N*-glycan carrier protein, GlycoTag, given parenterally [104].

## 6. Multi-Pathogen Strategies

Protective and relatively conserved antigen candidates are certainly available for each of the three targeted enteric pathogens, but at present, relatively little effort is being made to consolidate them into a single multi-pathogen vaccine. Efforts made to date have been directed towards the combination of ETEC and *Shigella* with the omission of *Campylobacter*. The inclusion of *Campylobacter* with these other pathogens could improve its value as a licensed vaccine. The consideration of combination vaccine approaches for these major causes of morbidity and mortality among infants and young children in LMIC settings, as well as among international travelers, have long been encouraged by donors and international stakeholders such as WHO and Gavi [105,106,107]. It has only been recently that candidate antigens, adjuvants, and formulation strategies have existed that might make the pursuit of a multi-pathogen vaccine more feasible. An additional driver for this approach is the growing acceptance that a combination vaccine would have a more favorable full vaccine value assessment, which would help ensure a greater potential for wider uptake once available. In this vein, the WHO’s recent PPCs for both *Shigella* and ETEC vaccines also suggested that the combination vaccine approach for these pathogens should be explored [108].

### 6.1. Multi-Pathogen Vaccines for Oral Administration

The most active area for the consolidation of enteric pathogen vaccines is orally delivered whole cell vaccine candidates, some of which exploit relatively conserved antigens in addition to serotype-specific immunity. The utilization of oral immunization avoids possible problems associated with the multiple use of needles on a crowded EPI schedule in LMICs, as needed for parenteral administration [109]. The oral route could also avoid the challenge of having all of the components co-formulated in a single container for licensure, which could be associated with subunit injectable vaccines if developed independently, as oral vaccine can be co-administered rather than co-formulated, although the latter would be ideal and would benefit the full value proposition for the vaccine. On the other hand, the reduced efficacy of orally administered vaccines in LMIC settings compared with high-income countries has been clearly documented for rotavirus and cholera vaccines; therefore, applying this approach for an oral vaccine should be done cautiously and with a detailed plan to evaluate the interference between antigens (or vaccines) and mucosal immune responses. Recent evidence in Bangladeshi children given the oral whole cell vaccine, ETVAX, indicate that the inclusion of the mucosal adjuvant dmLT may improve immune responsiveness to orally administered vaccines [54].

Multi-pathogen cellular approaches are now being developed, most using an attenuated oral *Shigella* cellular vaccine candidate as a platform (Table 4).

1. The guaBA *Shigella*−ETEC hybrid 1208-122 should provide effective coverage against both *Shigella* and ETEC, relying on antigens that have been well established to protect [57]. A prototype of this candidate, *S. flexneri* 2a expressing CFA/I and LT, protected orally challenged mice against ETEC and *Shigella* [58], and may enter Phase 1 evaluation in 2021 (https://clinicaltrials.gov/ct2/show/NCT04634513, accessed on 1 May 2021). This approach demonstrates the use of expression of heterologous antigens to broaden coverage, which could also be applied to conserved *Campylobacter* antigens.
2. ShigETEC, in contrast with the hybrid, relies entirely on little studied but promising conserved surface proteins minus those of the deleted T3SS to protect against *Shigella* [49,50]. The strain contains a RfbF deletion that renders it rough, i.e., not expressing the serotype-determining O-PS. ETEC coverage is anticipated from homology existing between *Shigella* and ETEC [59,60], as well as the LT/ST chimeric toxoid expressed [51]. As stated above, the extent that the homology present in *Shigella* will contribute to protection against major ETEC strains remains to be determined.
3. A third platform at an earlier stage of development than the previous two is the *Shigella* truncated mutant (STM) comprised of inactivated *Shigella* mutants with O-polysaccharide chains truncated to one repeating unit in length [48]. This, like the ShigETEC, enhances the immunological accessibility of conserved and protective *Shigella* outer membrane proteins such as PSSP-1 [46], and may also be a benefit to vectored antigens. STM also express additional conserved proteins not normally masked by O-polysaccharide chains, such as the Ipa proteins (deleted in the ShigETEC approach), which could also contribute to broader serotype-independent immunity [36,37]. STM should also benefit from homology to ETEC, but it remains to be seen whether it may also be necessary to engineer the expression of some additional ETEC antigens such as EtpA or CS6 into the STM for optimal coverage. For instance, data emerging from the analysis of more than 1500 phylogenetically and geographically diverse isolates of ETEC suggest that a combination of CS6 and EtpA, which is more frequently absent in CS6-expressing strains, would afford coverage for more than 80% of ETEC [65,72]. The STM is the only candidate currently planned to be engineered to express the conserved *Campylobacter* heptasaccharide [104].
4. A recombinant live oral ETEC vaccine (Ty21a-ETEC) composed of Ty21a, the oral typhoid vaccine, expressing both heat-labile (LT) and heat stable enterotoxin (STa) and seven adhesins (CFA/I, CFA/II (CS1-CS3), and CFA/IV (CS4-CS6)) that facilitate the colonization of host intestines and binds GM1was constructed [110]. The seven adhesins comprise a multi-epitope fusion antigen (MEFA) that has shown to have a broad spectrum anti-adhesin activity. The intranasal (IN) immunization of BALB/c mice, which mimicked a mucosal/oral route of immunization, induced antibodies against LTB and MEFA that blocked binding to GM1, showing the induction of an anti-toxin activity. Further antibodies induced by IN immunization induced antibodies that blocked the adhesion of ETEC to Caco-2 cells. The typhoid vaccine has also served as a vector for the major *Shigella* O-PS [20,21].

### 6.2. Multi-Pathogen Vaccines for Parenteral Administration

In contrast to the current activity with the cellular candidates, injectable subunit candidates addressing multiple enteric pathogens have been minimally pursued. Currently, the promising subunit vaccine candidates described above are being developed as single pathogen products that could add a likely unacceptable number of injections to an already crowded immunization schedule. If developed separately, consolidation could be a long and expensive process. On the other hand, parenteral administration of a multi-pathogen vaccine could avoid issues with reduced vaccine efficacy in LMIC populations that have plagued oral rotavirus and cholera vaccines and reduce the number of injections required. Recently, one group has described a prototype for a tri-pathogen subunit conjugate vaccine to cover *Shigella*, ETEC, and *Campylobacter* [111]. This vaccine could consist of eight *C. jejuni* CPS, four *Shigella* O-PS, and four to five ETEC adhesin proteins. This approach is intriguing; however, valency requirements may be problematic. A more efficient strategy could be to exploit the more broadly conserved *C. jejuni* antigens (e.g., heptasaccharide or FlaA); however, questions remain regarding their potential to serve as human vaccine antigens.

**Figure 2:**
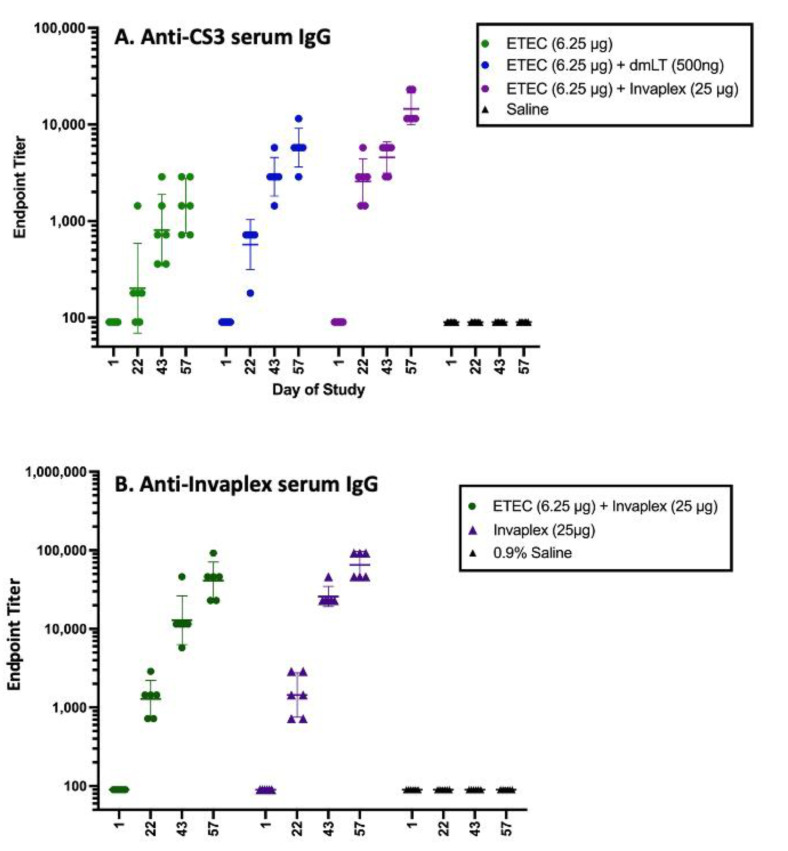
Immune response to *Shigella* and ETEC CS3 antigens in a combined vaccine as shown in Figure 3. CS6 and CFA/I in combination with dmLT or Invaplex had significantly higher anti-CS3 antibody titers compared with titers after immunization with CS3, CS6, and CFA/I alone (Panel A). The immune response to CS6 was similarly enhanced, but the immune response to CFA/I was unaffected. Serum IgG titers directed in Invaplex (Panel B) were comparable in guinea pigs immunized with CS3, CS6, and CFA/I delivered alone or in combination with Invaplex, indicating that the ETEC antigens did not interfere with the *Shigella* antigen-specific immune responses. For these unpublished data (R. Kaminski), Guinea pigs (Hartley strain; six pigs/grp) were immunized intradermally on study days 1, 22, and 43 with either 6.25 µg of CFA/I, CS3, and CS6 delivered with and without dmLT (500 ng) or Invaplex (25 µg). Blood collected on days 1, 22, 43, and 57 were assayed by ELISA for serum IgG titers directed to CS3 (**A**) and Invaplex (**B**). Data represents the geometric mean titer and 95% confidence interval.

In addition to its potential use as a *Shigella* vaccine, as it is capable of inducing broad immune responses across multiple *Shigella* serotypes, Invaplex has been shown to be an effective adjuvant, augmenting the immune responses directed to co-administered protein- or plasmid-DNA-based vaccines [112,113,114,115]. The adjuvant activity of Invaplex is attributed to the biological activity of the complex, which facilitates the cellular uptake of Invaplex and heterologous antigens, and the LPS component or Lipid A moiety that provides a “danger signal” to the immune response, resulting in effective immune processing and increased immunogenicity. Interestingly, the under-acylated Lipid A molecule utilized in Invaplex_AR-DETOX_ is also capable of providing a danger signal for enhanced immune responses, albeit at lower but more controlled levels. Using ovalbumin (OVA) as a model antigen, intranasal immunization with OVA combined with Invaplex was found to enhance the anti-OVA serum immunoglobulin G (IgG) and IgA responses and induce OVA-specific mucosal antibody responses at sites located both proximal and distal to the immunization site [112]. Subsequently, the adjuvant effect of Invaplex has been demonstrated for ETEC antigens CS3 and CS6 (Figure 2), as well as *Campylobacter* FlaA [115], in the absence of immune interference. These data suggest that Invaplex_AR-Detox_ can serve as a platform for the delivery of heterologous antigens to be included in a multi-pathogen vaccine, while still inducing potent immunity against shigellosis.

## 7. Considerations to Optimize the Immunological and Practical Impact of Vaccine Candidates to Provide an Effective Multi-Pathogen Vaccine Strategy

### 7.1. Pediatric Presentation for Oral Vaccines

**Figure 3:**
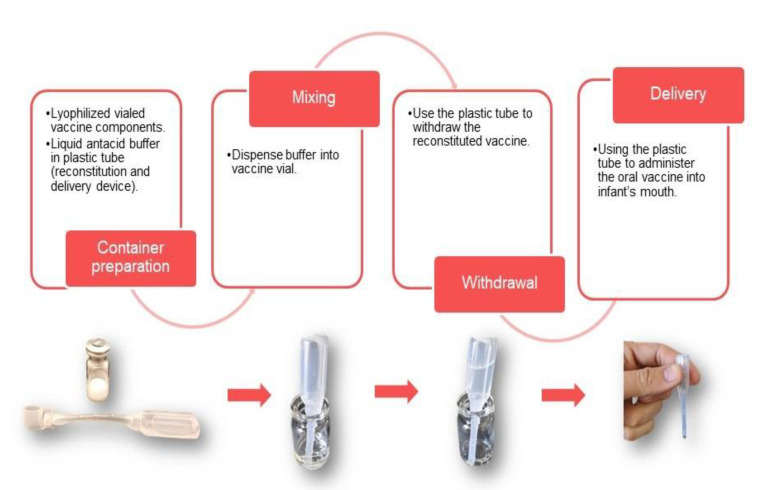
Presentation options for oral enteric vaccine delivery. Oral vaccine presentations can be made up of a combination of dry and liquid vaccine components that can be combined as shown.

While the tri-pathogen vaccine approach provides the benefit of protection against multiple pathogens in a single vaccine formulation of multiple antigens, the antacid buffer and, potentially, the adjuvant can be challenging. A currently licensed vaccine against cholera (Euvichol) may serve as a model for the packaging and delivery of a tri-pathogen vaccine. The inactivated whole cells in this vaccine are suspended in saline contained in a flexible plastic tube that can be used to deliver the vaccine directly to the mouth. This presentation allows for flexibility for the tri-pathogen vaccine final formulation; if, for example, vaccine components are found to be incompatible, they can be envisioned, as necessary, in separate flexible tubes or lyophilized in vials for co-administration (Figure 3). A buffer, for example, could be packaged in a separate tube, minimizing the vaccine antigens’ exposure to high osmolarity or lyophilized for an improved shelf-life of the final product [116,117,118,119]. To accomplish this for pediatric use, a citrate buffer similar to that being used with licensed oral rotavirus vaccines could be considered to avoid the rehydration process associated with the use of bicarbonate buffer vaccines and provide the needed shelf life in a liquid formulation. Similarly, for inactivated or live attenuated cells, lyophilization offers a means to achieve better long-term stability. Using this approach, lyophilized whole cells could be rehydrated using the liquid buffer through a modified flexible tube similar to a pipette. Once resuspended, the same flexible tube could be used for the subsequent administration (Figure 3).

### 7.2. dmLT Adjuvant

Whether an oral or intramuscular vaccine is developed, accumulating animal and clinical data are demonstrating the importance of considering use of the adjuvant dmLT with vaccines against mucosal pathogens. Mucosal and systemic immune responses to both live and inactivated vaccines can be improved by adding the adjuvant dmLT, a highly attenuated form of the ETEC heat-labile toxin (LT), to candidate vaccine formulations, while at the same time inducing anti-LT immunity [120,121,122]. Accumulating data have shown that dmLT may improve vaccine Th1/Th2/TH17 balance and enhance mucosal aspects of immune response in order to provide better protection by novel and established vaccines [122,123,124,125]. In fact, new adjuvant data from 6- to 11-month-old Bangladeshi infants, a difficult population in which to achieve robust immunity following the administration of vaccines, show that dmLT improves the frequency and the magnitude of the mucosal immune response to ETEC antigens following immunization with a killed whole cell vaccine, ETVAX [54]. Furthermore, in Bangladeshi infants, the kinetics of the mucosal immune response were accelerated by the inclusion of dmLT in the vaccine [123]. In addition, these studies in infants in Bangladesh have also shown that the dmLT adjuvant can improve the mucosal antibody response to both protein and polysaccharide antigens, and that the use of dmLT was particularly important for ETEC vaccine “take” in 6- to 11-month-old infants, as fractional doses of the vaccine needed to be used to improve its tolerability [35]. In earlier work, the live attenuated ETEC candidate vaccine ACE527 provided significant protection in a Phase 2b challenge study only when the vaccine was co-administered with dmLT [52]. Recent Phase 1 studies with prototype ETEC vaccine subunit antigens have shown that dmLT can be used safely through the intradermal and intramuscular routes, and that it can improve the frequency and magnitude of serum and mucosal antibody responses to these subunit ETEC vaccine antigens, CfaE and CssBA [64,66,126]. Based on results such as these, for both oral and parenteral vaccines, it is likely that the immunogenicity of a tri-pathogen vaccine in target populations may similarly be enhanced by the co-administration of dmLT.

### 7.3. Improved Tolerability and Immunogenicity of Oral Inactivated Whole Cell Vaccines for Infants

Recent clinical trials with ETVAX clearly demonstrate that safety, immunogenicity, and protection can be obtained with inactivated whole cell vaccines [54,55]. As has been found for the licensed inactivated cholera vaccine, Euvichol, standardization of vaccine dose for such vaccines based on the antigen content of the cells offers more effective immunization dosage control [127,128,129]. Although immunogenic, it has been found in infants that adult doses of ETVAX can induce mild vomiting in some children within hours of administration of an adult dose of vaccine, but fractional dosing (e.g., 1/8 to 1/4 of an adult dose) can reduce this tolerability issue without sacrificing immunogenicity [54]. This process reduces the levels of reactogenic LPS. Reactogenicity to LPS may, in the future, be reduced by the insertion of an msbB mutation in the LPS of the *Shigella* platform to under acetylate the lipid A of the endotoxin cell wall component [130]. A combination of pediatric doses and the msbB mutation in an endotoxin-containing preparation could help further resolve this tolerability issue. 

### 7.4. Dosing Schedule

As has already been indicated, the tri-pathogen vaccination strategy is directed at infants, the target population with the greatest need. To be accepted and practical in an LMIC, it would be important to be able to give multiple doses of vaccine on an existing vaccination schedule, thereby benefitting the value proposition for the vaccine. It has been reported that oral immunization with non-living antigens such as inactivated whole cells requires several doses to be given [131]. The gut IgA system needs this repeated exposure to achieve the multiplication of high-affinity B cell populations in the lamina propria. It is not known if vaccines given intramuscularly would require a similar kinetics as orally administered vaccines. The dosing schedule is important because some components of a multi-pathogen vaccine may be most important at different ages. Some of the target enteric pathogens, such as *Shigella*, may become more problematic later in life than others. With this in mind, a booster with the multi-pathogen vaccine at 9 months of age when the measles vaccine is given could be considered for priming doses given earlier. Experience with ETVAX in Swedish adults clearly indicates the possible benefit of an oral booster dose [132].

### 7.5. Animal Models for Protection

Even with the progress that has been made toward the development of effective antigens, moving to successful clinical development of a tri-pathogen product will benefit from the availability of predictive animal models of each disease for the numerous preclinical studies required. Although some models are available, this deficiency has been a hurdle for all three of the pathogens discussed in this review. New models have recently been reported that may improve the situation and greatly benefit the field. There is a high probability now that mouse models using nutrient deficiencies and/or antibiotic treatment to increase the susceptibility of animals to enteric infections recently developed at the University of Virginia may provide a simple and inexpensive way forward to test protection against intestinal disease following immunization and subsequent oral challenge with either of the three enteric pathogens of interest [133,134,135]. These models provide the opportunity to test a realistic oral infection against all three pathogens in the same small animal system. An alternate, complementary non-human primate model, *Aotus nancymaae*, provides a large-animal model, that, as with mice, can be infected orally with the development of subsequent disease manifestations from *Shigella,* ETEC, and *Campylobacter* [136,137,138]. 

### 7.6. CHIMs for Enteric Vaccine Development

The importance of CHIM studies for enteric pathogens has been clearly established by the experience with typhoid and cholera vaccines. In the case of the typhoid conjugate vaccine, a positive result in a CHIM study of healthy British adults was critical in augmenting Phase 3 field-study data for endemic countries such as Nepal [139,140,141]. The cholera vaccine Vaxchora^®^ was the first vaccine licensed by the US Food and Drug Administration, primarily on the basis of efficacy in a CHIM study [142]. However, Vaxchora was approved for travelers to cholera-endemic regions and additional data iares needed to establish its efficacy in LMIC residents.

CHIMs have been established for all three target pathogens [52,143,144,145]. CHIM studies have the potential to play a crucial role in the testing of vaccine candidates for these three pathogens for downselection and de-risking advancement to expensive Phase 3 field studies. The *Shigella* CHIM has a key role to play in the development of vaccines to prevent shigellosis. Several of the most advanced candidates have been tested in CHIM studies, including Flexyn2a [143] and GSK3536852A [144]. The results of these trials are drivers of the decisions of whether to advance one of these candidates to Phase 3 field studies. Nearly all *Shigella* CHIM studies have been conducted in US volunteers that presumably had little to no previous natural exposure to *Shigella*, and in some cases were pre-screened to ensure they were immunologically naïve. To address this potential limitation in relevance, efforts are underway to establish the *Shigella* CHIM in a low-income country setting [146]. 

Like *Shigella*, the ETEC CHIM is also expected to play a key role in vaccine development. The ETEC CHIM was used to demonstrate efficacy of the oral, live attenuated ACE527 candidate adjuvanted with dmLT [52]. Alternatively, other ETEC candidates such as ETVAX have progressed to field studies without first being tested in CHIM studies [54]. Future candidates will likely balance the comparative speed and efficiency of evaluation in a CHIM study, with the benefit of demonstrating efficacy in a more costly and complicated field study. For organizations or funders interested in downselecting from a portfolio of candidates, an ETEC CHIM study may be an attractive option.

The *Campylobacter* CHIM has been redeveloped because of safety concerns with the earlier 81-176 strain, which expresses ganglioside 2 and ganglioside 3, which have been epidemiologically linked to Guillain-Barré Syndrome [147]. The current model uses CG8421, which has no ability to express any ganglioside mimicry [145]. In one study of 15 subjects challenged with this organism, 93% subsequently experienced campylobacterosis. The CG8421 CHIM also provided a high attack rate in a rifaximin prophylaxis trial [148] and in an unpublished vaccine trial with ACE393 (NCT00859716).

## 8. Conclusions

Approaches to vaccines against enteric pathogens have been complicated not only by the numerous virulence factors, serotypes, and species involved, but also by the challenge of achieving protective immunity in the highest risk pediatric age-groups, the variety of vaccine formats (i.e., whole cell or subunit), and routes of administration. Several combinations of antigens are now available that could be effective for inducing protective immunity against each of the three highly prevalent target pathogens. The challenge is to achieve a combination of protective antigens with relatively few components so that the vaccine will be efficiently developed and will be cost-effective for subsequent manufacture.

The product achieved should ideally be optimal for the pediatric population initially and should capitalize on conserved antigens effectively delivered in an LMIC setting. Although the pediatric population is a major target for enteric vaccines, the travelers’ market may provide additional economic incentive to help drive vaccine manufacture. The value proposition for enteric vaccines emphasizes the importance of a vaccine to cover more than one pathogen [107,108]. Based on the state of the art presented here, it seems likely that this can be achieved through the development of both orally and parenterally administered candidates. However, as discussed above, a unified approach to a tri-pathogen subunit vaccine would be valuable, justified, and feasible. Regardless of whether enteric vaccines are for administration orally or parenterally, serious consideration should be given to the inclusion of an adjuvant like dmLT to help promote a protective mucosal response and provide dose sparing.

Decades have passed without obtaining licensure for vaccines against *Shigella, Campylobacter,* or ETEC. We now have tools and knowledge not previously available. The field is in a strong position to soon develop vaccines for *Shigella* and other enteric pathogens in order to enable a successful multi-pathogen vaccination strategy. However, this goal is in jeopardy because of the limited number of major donors in the field, but it is possible that new donors and manufacturing partners can be found to help offset this problem. With timely, sufficient, and sustained funding, knowledge and tools are now available to make a multi-pathogen enteric vaccine a reality. Early involvement of a manufacturing partner in this process should further ensure the realization of the opportunities now before us. The benefits of a tri-pathogen enteric vaccine to those living in LMICs are too great to let the current opportunity pass.

## Figures and Tables

**Table 1:** *Shigella* vaccine candidates (in development or previously found efficacious).

Candidate Name [Developer]	Pre Clinical	Ph1	Ph2	Ph3
**Inactivated cellular candidates**				
Truncated mutant (International Vaccine Institute (IVI), PATH)	X			
Trivalent *Shigella* whole cell (PATH, Walter Reed Army Institute of Research (WRAIR))		X		
Inactivated *Shigella* (Hilleman Laboratories)	X			
**Live attenuated cellular candidates**				
GuaBA-based live attenuated (CVD1208S; University of Maryland, Baltimore (UMB), PATH)		X		
VirG-based live attenuated (WRSS1, WRSs2, WRSs3, and WRSf3; WRAIR, PATH)			X	
Ty21a typhoid vaccine expressing *Shigella* LPS (Protein Potential)	X			
*Shig*ETEC (Eveliqure)	X			
**Glycoconjugate candidates**				
Chemically prepared glycoconjugate (National Institute of Child Health and Human Development)				X
Recombinant glycoconjugate (LimmaTech)			X	
Synthetic glycoconjugate (Institut Pasteur)		X		
**Subunit candidates**				
Invaplex_AR-DETOX_ (WRAIR, PATH)		X		
Generalized Module for Membrane Antigens (GMMA; GSK Vaccine Institute for Global Health)			X	
Outer Membrane Vesicles (OMV; University of Navarra)	X			
Ipa DB Fusion (PATH, University of Kansas)	X			
IpaB—GroEL fusion (Defense Inst. of Physiology and Allied Sciences)	X			
34kDa OMP (National Institute of Cholera and Enteric Diseases)	X			

**Table 2:** Enterotoxigenic *Escherichia coli* (ETEC) vaccine candidates (in development or previously found efficacious).

Candidate Name (Developer)	Pre Clinical	Ph1	Ph2	Ph3
**Inactivated cellular candidates**				
ETVAX inactivated (Scandinavian Biopharma, University of Gothenburg, PATH)			X	
STM (IVI, University of Georgia (UGA), PATH, Washington University (WASHU), WRAIR)	X			
**Live attenuated cellular candidates**				
ACE527 live attenuated (PATH; National Vaccine and Serum Institute, UGA)			X	
ShigETEC (Eveliqure)	X			
CVD GuaBA *Shigella*−ETEC hybrid (UMB, Emergent)	X			
Ty21a expressing *Shigella* LPS and MEFA (Protein Potential)	X			
**Subunit and toxin candidates**				
FTA (PATH, Naval Medical Research Center (NMRC), Sanofi, IDRI)		X		
MEFA (Kansas State University, John Hopkins University, PATH)	X			
LT/ST Fusion/conjugate (ENTVAC Consortium, PATH)	X			
dmLT mucosal adjuvant and antigen (PATH, Tulane)			X	
Flagellin, EtpA, EatA, EaeH, and YghJ (WASHU)	X			

**Table 3:** *Campylobacter* vaccine candidates (in development or previously found efficacious).

Candidate Name [Developer]	Pre Clinical	Ph1	Ph2	Ph3
**Inactivated cellular candidates**				
STM—heptasaccharide (IVI, PATH, UGA, and WRAIR)	X			
ACE527 expressing heptasaccharide (PATH, UGA, University of Alberta (UA))	X			
**Subunit candidates**				
Capsular Polysaccharide (NMRC)		X		
*N*-glycan Heptasaccharide (UGA, UA, PATH)	X			
Truncated flagellin FlaA-MBP (NMRC)	X			
B-subunit of cholera toxin (Kuwait University)	X			

**Table 4:** Multi-pathogen vaccine candidates.

Route of Administration	Current Description	Status	Possible Added Pathogen Antigens
**Oral Route**			
**Live attenuated [developer]**			
CVD GuaBA *Shigella*-ETEC hybrid (UMB, Emergent)	Major O-PS *Shigella* serotypes expressing CFA/I and CS antigens as well as LTB and ST toxoid of ETEC	Prototype nearing Phase 1	***Campylobacter*:** conserved antigen such as Heptasaccharide or FlaA
ShigETEC (Eveliqure)	O-PS and T3SS-free *S. flexneri* 2a expressing ST and LT toxoids to cover ETEC along with *Shigella* antigens with homology to ETEC	Nearing Phase 1	***Campylobacter*:** conserved antigen such as Heptasaccharide FlaA
Ty21a expressing *Shigella* LPS and MEFA (Protein Potential)	Ty21a expressing *Shigella* LPS and ETEC colonization antigens as a MEFA	Preclinical	***Campylobacter*:** conserved antigen such as Heptasaccharide or FlaA; ***Shigella*:** T3SS (Ipa)
**Inactivated cells [developer]**			
Truncated *Shigella* mutant (IVI, PATH)	wzy mutant (O-PS side chain shortened to one repeating unit) of Sf2a + ETEC antigen homology + dmLT	Preclinical	**ETEC:** ETVAX; non-canonical ETEC antigens (i.e., EtpA, CssBA or YghJ) ***Campylobacter*:** Heptasaccharide or FlaA
**Parenteral Route**			
**Subunit (developer**			
Tri-pathogen conjugate (NMRC)	*Shigella* O-PS + ETEC adhesin proteins + *Campylobacter* CPS	Preclinical	***Campylobacter*:** Heptasaccharide or FlaA to replace CPS; more conserved antigens could be included for **ETEC and *Shigella***
Invaplex (WRAIR, PATH)	IpaB + IpaC + *Shigella* LPS with msbB mutation	Prototype Phase 1 completed	**ETEC:** CFA/I, CS3, CS6 and/or ETEC non canonical proteins ***Shigella:*** additional LPS serotypes ***Campylobacter*:** Heptasaccharide or FlaA

## Disclaimer

Material has been reviewed by the Walter Reed Army Institute of Research and the Naval Medical Research Center. There are no objections to its presentation and/or publication. The opinions or assertions contained herein are the private views of the author, and are not to be construed as official, or as reflecting the true views of the Department of the Army, Department of the Navy, or the Department of Defense. The investigators adhered to the policies of protection of human subjects as prescribed in AR 70–25.

## Copyright Statement

Some authors (C.P. and R.W.K.) are employees of the US Government. This work was prepared as part of their official duties. Title 17 U.S.C. §105 provides that “Copyright protection under this title is not available for any work of the United Sates Government”. Title 17 U.S.C. §101 defines a US Government work as a work prepared by a military service member or employee of the US Government as part of that person’s official duties.
